# Effect of *Beta vulgaris* L. as feed ingredient on muscle growth, nutritional factors, and quality of common carp, *Cyprinus carpio*

**DOI:** 10.1002/fsn3.3496

**Published:** 2023-06-09

**Authors:** Nahid Amiri, Ali Aberoumand, Saeed Ziaei‐nejad

**Affiliations:** ^1^ Department of Fisheries Behbahan Khatam Alanbia University of Technology Behbahan Iran; ^2^ Department of Fisheries, Natural Resources College Behbahan Khatam Alanbia University of Technology Behbahan Iran

**Keywords:** *Beta vulgaris* leaf powder, carp fish, feed ingredient, nutrition, proximate composition

## Abstract

The purpose of this study was to evaluate the utilization of *Beta vulgaris* powder as feed ingredient in the diet of *Cyprinus carpio* for a period of 56 days. Common carp fry with an average weight of 20 ± 0.2 g were fed using the diet containing *B. vulgaris* leaves with different concentrations. Fry of *Cyprinus carpio* were equally distributed in four feeding groups having three replicates each. The study was conducted indoors, in FRP tanks, and aeration was provided to individual rearing units, and it was a flow‐through system. The basal diet was replaced at 0.5%, 1%, and 2% with *B. vulgaris* powder. The basal diet without *B. vulgaris* powder (0%) served as control (T1). Significantly higher feed conversion ratio, protein efficiency ratio, and fat and protein indices were recorded in fish fed with *B. vulgaris* powder in treatments. Different feeding groups showed greater acceptability of *B. vulgaris* powder mixed diet without any adverse behavioral response. The protein and fat average percentages were 43.32 and 10.79 g, when fish reached commercial weight (48.02, 11.85 g) after 56 days for treatment 2%. After fish feeding with the *B. vulgaris*, for treatment 2% lower moisture than the control was recorded. The carp fish diet containing 1% *B. vulgaris* leaf powder caused a significant decrease in the fish fat content. It can be concluded that the diet containing 2% *B. vulgaris* leaf powder in the common carp led to better growth performance. The presence of *B. vulgaris* leaves in the fish diet increased the fillet protein and ash content.

## Graphical abstract

Different feeding groups showed greater acceptability of *Beta vulgaris* powder mixed diet without any adverse behavioral response. The study was set up with protein and fat average percentages of 43.32 and 10.79 g and finished when fish reached commercial weight (48.02, 11.85 g) after 56 days for treatment 2%. After fish feeding with the *B. vulgaris*, lower moisture than the control was recorded. Adding 1% *B. vulgaris* leaf powder to the carp fish diet caused a significant decrease in the fish fat content. It may be concluded that 2% *B. vulgaris* powder may be used in the diet of common carp for better growth performance. The presence of *B. vulgaris* leaves in the fish diet increased the fillet protein and ash content.

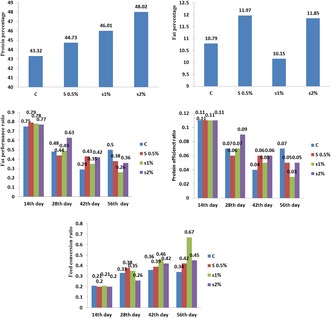

## 1 INTRODUCTION

Beetroot (*Beta vulgaris* L.) belongs to the Chenopodiaceae family and is originally from temperate climate regions of Asia. In Iran, it is grown in all regions. In markets, their leaves are cut off from the bulb to be used as organic fertilizer and animal feed. Beetroot leaves are underused due to a lack of proper knowledge, especially of their nutritive value and how to cook them and also because of dietary habits (Biondo et al., 2014). *Beta vulgaris* leaf powder is a rich source of nutrients. These nutrients include folic acid, vitamins A, C, and B_6_, niacin and biotin, and minerals like iron, magnesium, selenium, potassium, calcium, zinc, phosphorus, and sodium (Straus et al., 2012). Its pigments are natural, harmless, and useful in the food industry (Esatbeyoglu et al., 2015). Bioactive constituents include phenolic compounds of saponins, especially betaine (Zielinska‐Przyjemska et al., 2009). In recent years, red beetroot (*B. vulgaris* rubra) is used as a low‐cost, nontoxic supplement that promotes health (Clifford et al., 2015). Sugar beet has antiviral (Strack et al., 2003), antioxidant, and anti‐inflammatory properties (Georgiev et al., 2010). It also inhibits human tumor cell proliferation and promotes antioxidant activity (Lechner et al., 2010).

It has been reported that beet leaves are an excellent source of omega‐3 fatty acids in addition to having significant antioxidant activity and a huge amount of mineral phenolic compounds (Biondo et al., 2014). The chemical components in the leaves change during the developmental stages. *Beta vulgaris* leaf powder has the highest protein and lipid contents. As meals or add‐ons to other foods, the dried leaves have high nutritional value (Biondo et al., 2014).

During the first 100 days of growth, omega‐3 and omega‐6 fatty acids and some of phenolic compounds and other minerals are found in the fish body. Common carp is an omnivorous species and its food spectrum includes benthic organisms, insects, aquatic plants, and preyphones. However, the digestive system of carp is consistent with the formulated diet (Mráz, 2011). Common carp has the potential to efficiently utilize fat and carbohydrates as a source of energy (Takeuchi et al., 2002). Other benefits include rapid growth, maturation in the second year of life, extremely high fertility (two million eggs per female fish), and the ability to manually ovulate (Troca & Vieira, 2012). A combination of all these features has given this species high potential for aquaculture (Troca & Vieira, 2012). These factors have made this species grow rapidly throughout the world (Saikia & Das, 2009). *Beta vulgaris* L. is cultivated throughout the world because its roots are used as food and as a source of natural pigment. It is a rich source of important nutrients including magnesium, sodium, potassium, vitamin C, and betaine. Betaine (beet pigment) has been studied as natural colorant in food products (Rabeh, 2015), and a common additive in aqua foods. The purpose of this research was to investigate the effect of *B. vulgaris* leaf powder on the proximate composition and nutrition indices of common carp fish.

## 2 MATERIALS AND METHODS

This research has been done in 2016 at Behbahan Khatam Alanbia University of Technology, Iran for 6 weeks. For this purpose, 12 fiberglass cylindrical tanks (300 L) for treatments and two 500‐L tanks for water storage were used. The tanks were used for four treatments in triplicate. Aeration tubes were installed in each of the air stones and then placed inside each tank. Then, they were aerated for 48 h to remove urban water chlorine.

In the present study, young common carp fish with a weight of approximately 20–23 g and a relative length of 8–9 cm were considered. The 500 common carp fish were bought from Native Fish Resources, then the fish were transferred to the Fisheries laboratory at Behbahan Khatam Alanbia University of Technology, Iran. After dividing the fish into five bags, one third of each bag was filled with water, and the rest was filled with oxygen. At the beginning of the work, the bags containing fish were placed in a pre‐prepared tank so that the bags were temporarily filled with the reservoir's water; otherwise, the fish would be heat‐shocked and die. The existence of air pipes in the bags is necessary to prevent oxygen deficiency. After 2 h, the bags were slowly opened, and the fish were stored in the tanks filled with water.

With the arrival of the fish in the storage tank, the adaptation period began before the project. The length of the adaptation period was considered 1 week. In order to feed the fish during this period, a special basic diet for carp called Fara Daneh was purchased and used. *B. vulgaris* grown in South Khuzestan Province, Iran was purchased from a local market (Behbhaan, Iran). After sorting and cleaning step, whole leaves were dried at 65°C for 12 h in an industrial‐scale cabinet drier. *B. vulgaris* leaf powder was used as an ingredient in the formulation of the fish diets at levels of 0% (Control), 0.5% (T1), 1% (T2), and 2% (T3) of total content of component and were produced as extruded feed.

### 2.1 Preparation of fish diets

In order to add supplement to the diet, first the food was soaked in sterile water and then it was made into a paste. After that, the food supplement was added to the paste in the stated proportions, then it was completely mixed and homogenized, then the food was made into pellets with the appropriate size for the fish's mouth using a meat grinder, and the pellets were dried.

The level of feeding to the fish was three times a day and equal to 2% of the fish's body weight. In order to minimize the bacterial interactions in the prepared diet, the food was prepared daily and fed to the fish. The amount of daily food was calculated on a weekly based on the biomass for each tank separately. The feeding percentage was obtained according to the fish size and water temperature and based on the feeding table (Table 1).

**TABLE 1:** Diet formulation of the basal diet.

Ingredients	Percentage
Fish meal	30
Corn meal	20
Wheat flour	18
Soybean meal	16
Rice bran	8
Fish oil	2
Soybean oil	2
Mineral mix	2
Vitamin mix	2

### 2.2 Sampling and harvesting

The fish feeding was stopped for 48 h to empty the fish's digestive system, then six fish were randomly selected from each tank, and their weight and length were calculated. The liver and viscera were used to calculate the weight. Then, the skin was removed, and the fish tissue was separated to determine its chemical composition.

### 2.3 Experimental design

Three treatments depending on concentrations of *B. vulgaris* leaf powder in fish feed were used in this study where control (C) fish were fed with feed without *B. vulgaris* leaf powder and other treatment groups were fed by feed with 0.5% (T1), 1% (T2), and 2% (T3) *B. vulgaris* leaf powder.

### 2.4 The reviewed parameters

The reviewed parameters are presented below.

1. Condition factor (CF)
2. CF = Acquired weight (g) − [total length (cm)]/2 × 100
3. Nutritional indicators
4. Feed conversion ratio (FCR)
  FCR = Average diet (g)/Average fresh body weight gain (g)
5. Fat performance ratio
6. Protein efficiency ratio (PER)
7. PER = weight gained/weight of protein consumed.

### 2.5 Chemical composition analysis

The standard method (AOAC, 2005) was used to measure the proximate composition of the fish tissue samples. All experiments were performed in replicate. The proximate compounds were moisture, crude fat, crude protein, and crude ash. The samples were sent to the Central Laboratory of the Veterinary Office in Ahvaz for sample analysis. To powder the samples, 200 g of fillets was placed in an oven at 65°C for 24 h to be dried completely. The samples were then cooled down and powdered by the grinder. Then, they were put in separate containers, and finally, transferred to the laboratory for analysis.

### 2.6 Measurement of moisture

The moisture content was determined by drying 10 g of the powdered sample in the oven at 105°C for 24 h to reach a constant weight (AOAC, 2005). After removing the dish from the oven, it was placed in desiccator for 30 min to be cooled down. The sample was then weighed, and its moisture content was determined using the following formula:

$$\begin{array}{c} \text{Moisture content}\\ =\frac{\text{sample initial weight}\;\left(g\right)-\text{sample secondary weight}\;\left(g\right)\times 100}{{\text{sample}}^{’}s\;\text{initial weight}\;\left(g\right)}. \end{array}$$

### 2.7 Measurement of crude fat

Total fat content was determined by the Soxhlet method (James, 1995). The German scientist, Soxhlet, is the founder of the fat extract method, which was later used worldwide to extract lipids from biological materials. To do this, we put 5 g of homogenated sample into Erlenmeyer. Then, we added 35 mL of concentrated HCL and 6 mL of distilled water and heated it. Erlen content was filtered through the filter paper and then the filter paper was washed with hot water.

To dry the filter paper, we inserted it into the oven and cooled it down in the dishwasher. After drying, the fat was extracted using an ether solvent.

Then, the extraction balloons were separated from the apparatus and the solvent residue was evaporated using a water bath. Finally, to dry the balloon until reaching its final weight, it was placed in the oven, and then cooled down in the desiccator to obtain the total fat content of the sample. The fat content of the sample was determined using the following formula:

$$\begin{array}{c} \mathrm{Fat}\;\text{content}\\ =\frac{\text{balloon secondary weight}-\text{balloon primary weight}}{\text{sample weight}}\times 100. \end{array}$$

### 2.8 Measurement of crude protein

Protein assay was performed using the Kjeldahl method (James, 1995) and the Kjeldatherm apparatus. For this purpose, 1 g of the homogenated sample with 20 mL of concentrated sulfuric acid and 8 g of catalyst were placed in a special tube and transferred to a Kjeldahl digestive tract. Digestion was performed for 30 min at 250°C and for 45 min at 410°C. The sample was then placed in a distillation machine. Eighty milliliters of distilled water and 80 mL of 32% NaOH were added automatically to the sample. Distillation vapors were introduced into a 2% boric acid container with a few drops of reagent, and finally, titrated to 0.1 N HCl. The nitrogen content of the sample was calculated by the following formula and the obtained number is multiplied by 6.25 to determine the amount of protein:

Nitrogen content=HCLintake volume×1.4007×0.1.

### 2.9 Ash measurement

The ash content of the sample was determined by an electric furnace (AOAC, 2005); 10 g of dried sample was placed in a Chinese clay crucible. Then, it was placed in an electric oven at a temperature of 500–550°C until it turned gray. The residual material in the clay crucible indicates the amount of sample ash:

$$\begin{array}{c} \mathrm{Ash}\;\text{content}\\ =\frac{\text{initial dish}+\text{sample weight}-\text{secondary dish}+\text{sample weight}}{\text{sample weight}}\times 100. \end{array}$$

### 2.10 Data analysis

To analyze the data, the normal distribution of the data was determined using the SPSS software and the Kolmogorov–Smirnov test. Then, using one‐way ANOVA, the possible difference between the treatments was determined. In addition, after observing a significant difference, Duncan's test at 95% confidence level was used to check the difference between the treatments. The Excel software was used to draw the charts.

## 3 RESULTS AND DISCUSSION

**FIGURE 1:**
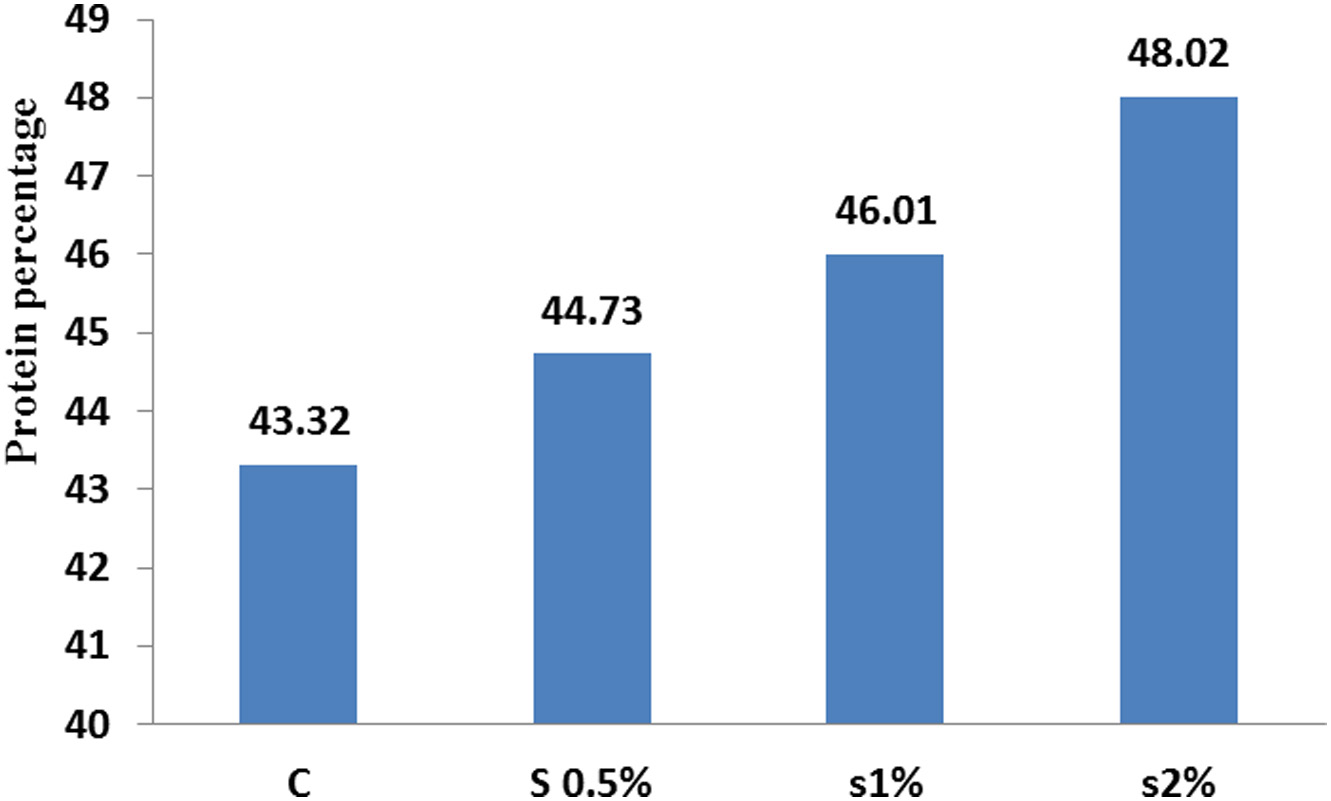
Protein index in different treatments compared to the control treatment. Similar letters in each column indicate no significant difference (*p* < .05).

**FIGURE 2:**
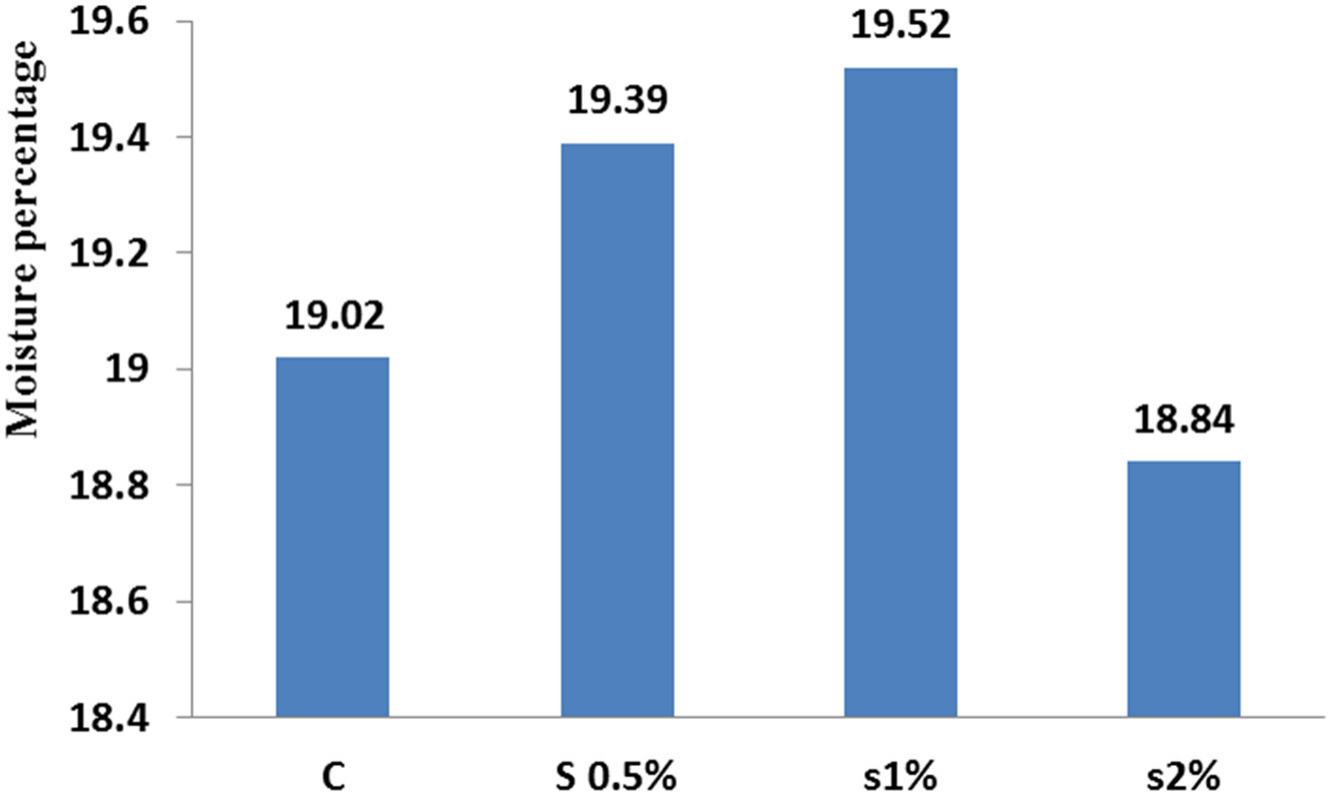
Moisture index in different treatments compared to the control treatment. Similar letters in each column indicate no significant difference (*p* < .05).

**FIGURE 3:**
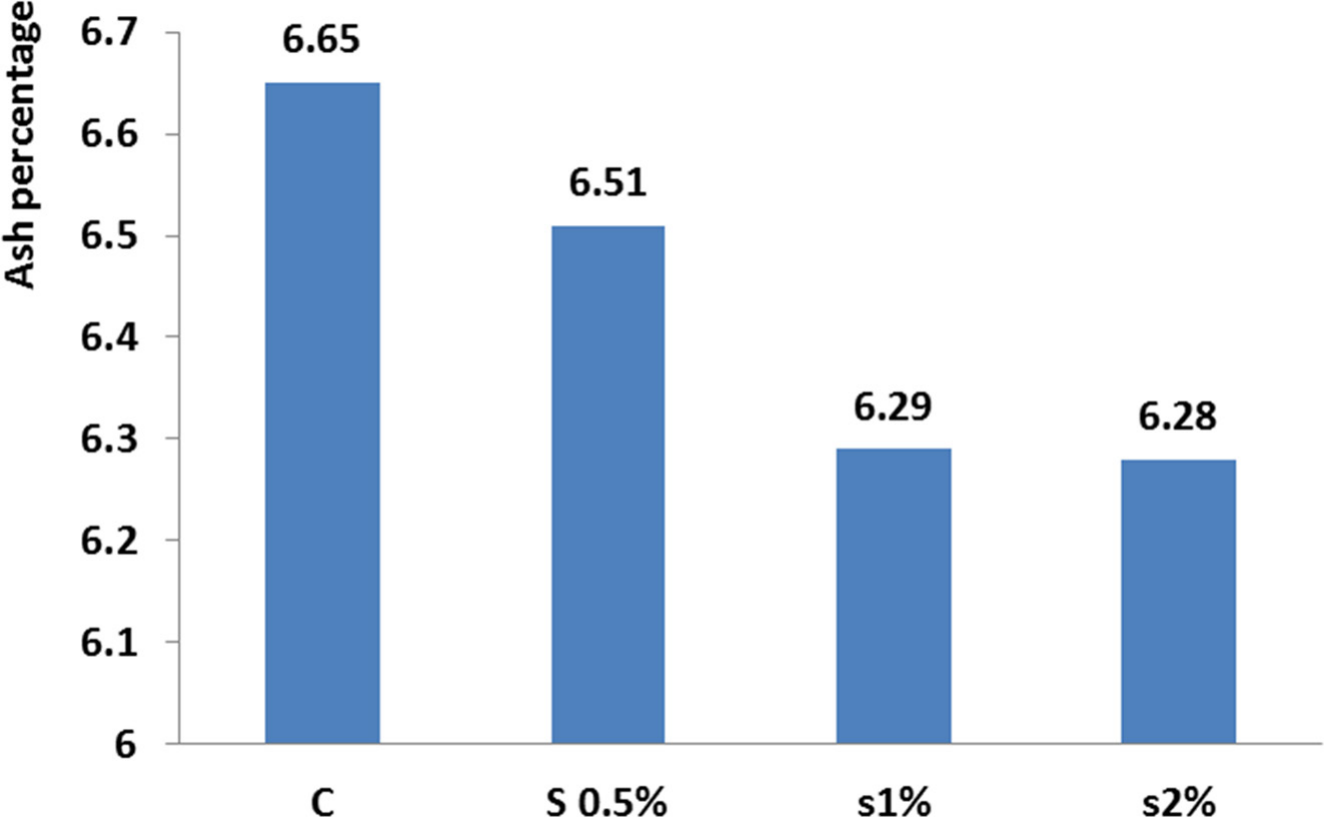
Ash index in different treatments compared to the control treatment. Similar letters in each column indicate no significant difference (*p* < .05).

**FIGURE 4:**
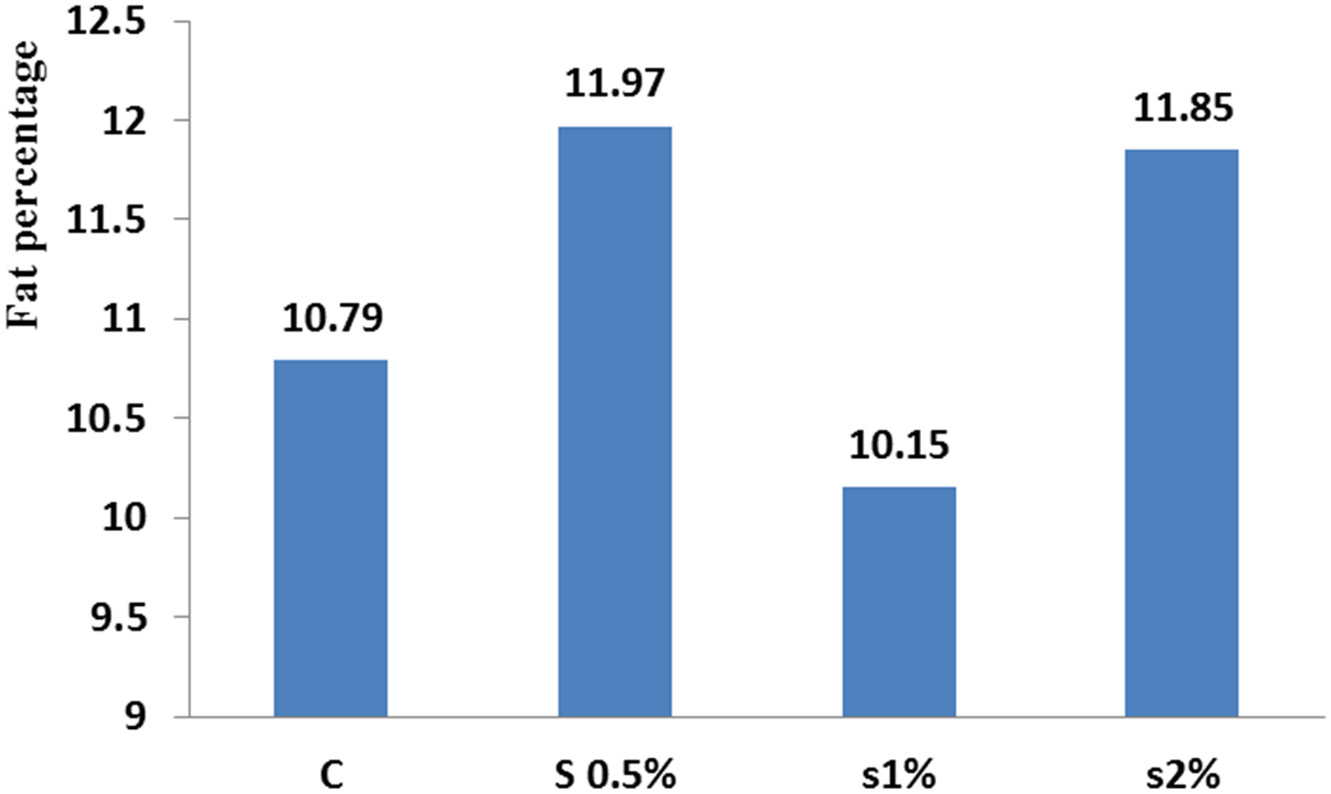
Fat index in different treatments compared to the control treatment. Similar letters in each column indicate no significant difference (*p* < .05).

**FIGURE 5:**
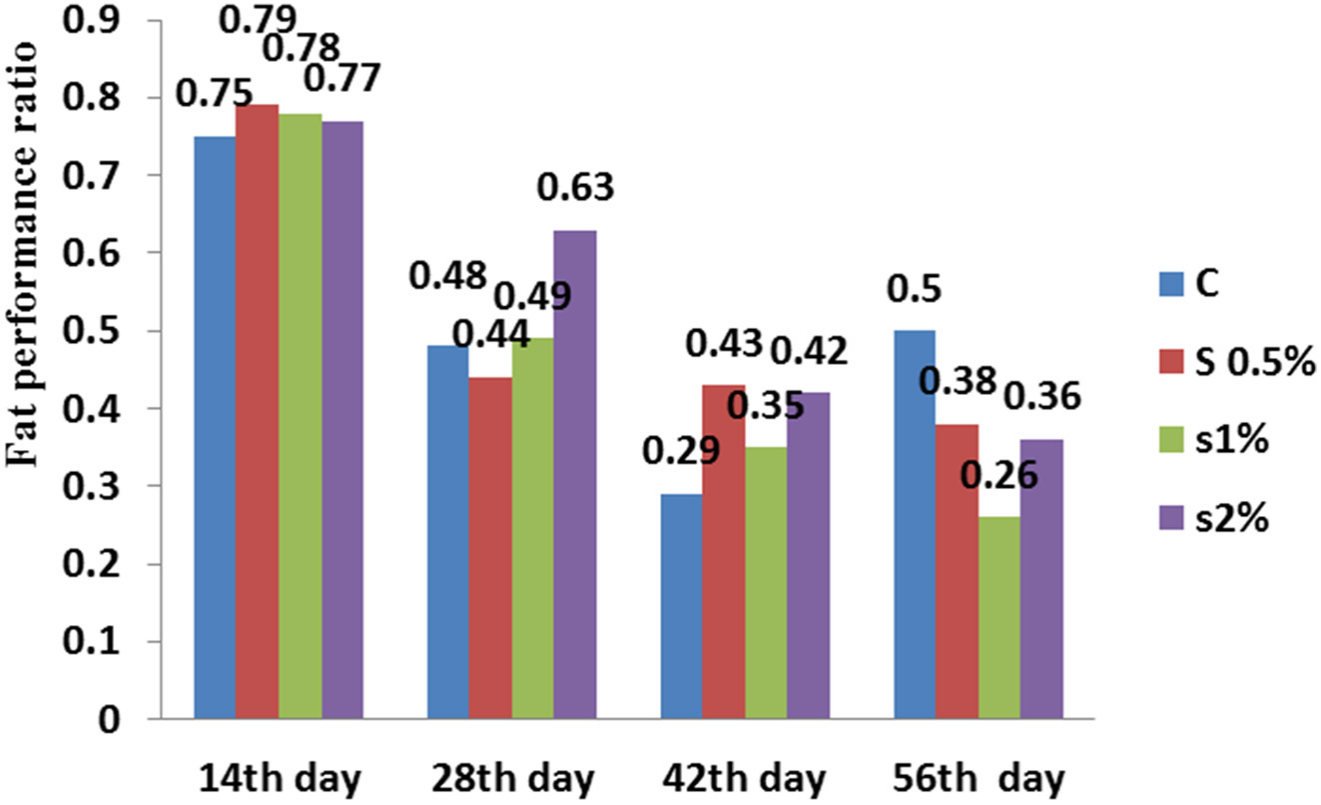
Fat performance ratio in different treatments compared to the control treatment. Similar letters in each column indicate no significant difference (*p* < .05).

**FIGURE 6:**
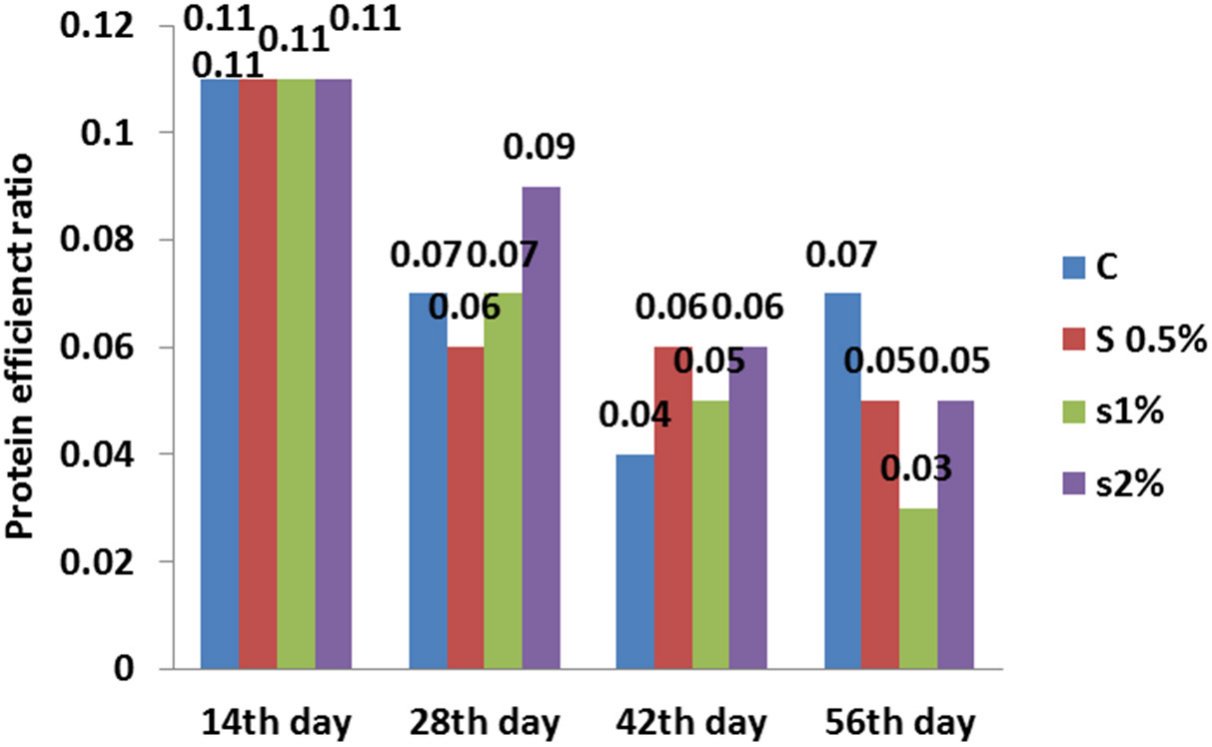
Protein efficiency ratio in different treatments compared to the control treatment. Similar letters in each column indicate no significant difference (*p* < .05).

**FIGURE 7:**
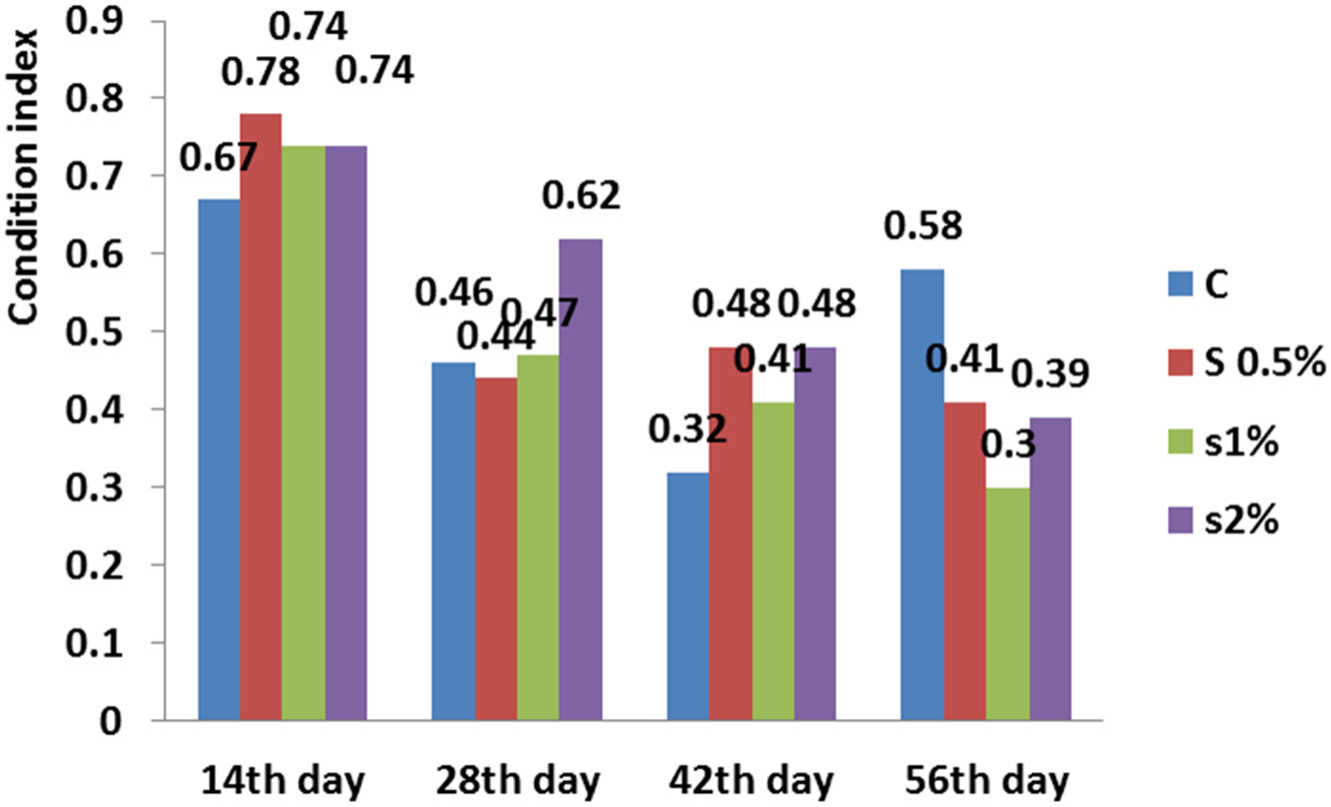
Condition index in different treatments compared to the control treatment. Similar letters in each column indicate no significant difference (*p* < .05).

**FIGURE 8:**
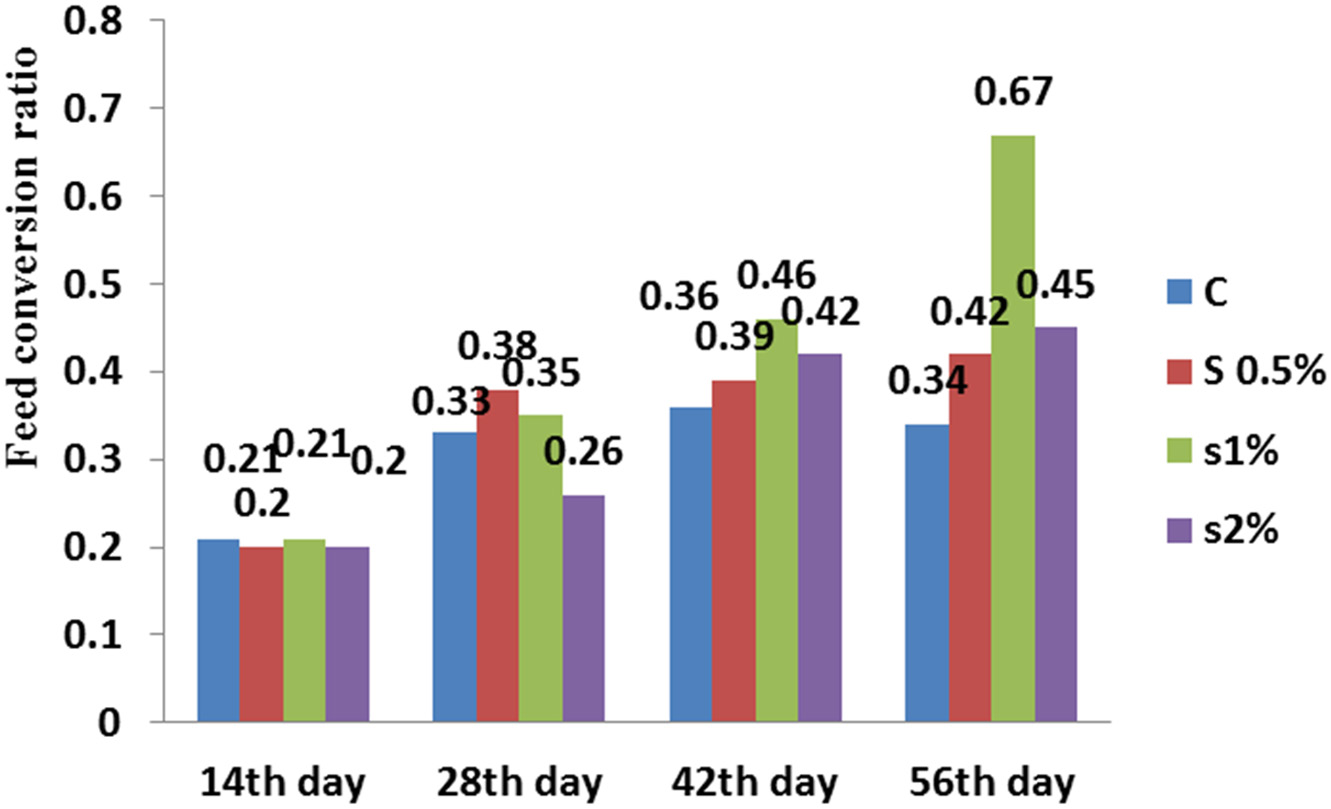
Feed conversion ratio in different treatments compared to the control treatment. Similar letters in each column indicate no significant difference (*p* < .05).

The obtained results are shown in Figures 1, 2, 3, 4, 5, 6, 7, 8. All data were obtained from the mean of triplicate tests. There was a significant difference between the treatments and control in the fish fillet protein content (*p* < .05; Figure 1). Adding *Beta vulgaris* leaf powder to the fish diet reduced their moisture content compared to the control treatment (*p* < .05; Figure 2).

For the ash index, there was a significant difference between the control treatment and 1% and 2% treatments, while significant difference was not observed between the control and 0.5% treatment (*p* < .05; Figure 3). For the fat index, there was a significant difference between the control treatment and the other treatments (*p* < .05; Figure 4). For fat performance ratio on the 46th day found a significant difference between the control and 1% treatment. There was no significant difference between 1% and 2% treatments (Figure 5; *p* < .05).

The protein efficiency ratio decreased for 1% treatment, but there was a significant difference compared to the control treatment (*p* < .05). The protein efficiency ratio was found same for 2% and 0.5% treatments, but lower than the control treatment on the 56th day (*p* < .05) (Figure 6).

There was a significant difference between the control and 1% treatment on the 56th day for condition index (*p* < .05; Figure 7). The feed conversion ratio in the treatments was significantly higher than in the control treatment. On the 56th day, the control treatment had the lowest and 1% treatment had the highest feed conversion ratio (*p* < .05; Figure 8).

This implies that a maximum concentration of 1% *B. vulgaris* leaves powder in fish nutrition is the most suitable option, while the present study result showed that the concentration of 2% of *B. vulgaris* leaf powder increased the fish fillet protein content to 48% (Figure 1). Results in Figure 4 showed that 0.5% and 2% concentrations of *B. vulgaris* leaf powder were able to synthesize fat in the fish body, but at 1% concentration, the fat content was decreased, implying that the nutritional and environmental conditions of fish have been affected.

Results in Figure 8 showed the feed conversion ratio in the concentration of 1% on the 56th day, had the highest, while in concentrations of 2% and 0.5% on the 14th, 28th, 42nd, and 56th days, this index did not show significant difference.

Protein content in the fish fillet was found 48.02% for the 2% concentration treatment, which was the highest yield compared to the other treatments as well as the control treatment. With increasing the *B. vulgaris* leaf powder concentration (maximum 1%), the moisture content in the fish fillet increased (19.5%), but in 2% concentration, the moisture content decreased (18.8%) (Figure 2). It was revealed that with increasing the concentration of *B. vulgaris* leaf powder, the protein content in the fish fillet increased, and the moisture content decreased to a concentration of 2%, due to increasing the WHC (water‐holding capacity) of protein in the fish fillet.

Pinedo‐Gil et al. (2017) reported that inclusion of 14% red beet *Beta vulgaris* did not affect growth, nutritional or biometric parameters as well as nutrient retention compared to the control, while higher red beet had a negative effect on growth and nutritional parameters, which was not agreed with present study results. The Pinedo‐Gil et al. (2017) results may be partly due to the effect of some antinutritional components in red beet such as tannins or oxalates, which reduce growth and can lead to poor FCR and PER.

However, dietary inclusion of red beet significantly reduced AD_EE_. This reduction may be related to changes in lipid and/or CHO metabolism pathways. The presence of oxalate and its ability to bind minerals in the intestine reduces the digestibility of fat. Also, this effect can be related to the higher VSI and HSI found after feeding with higher ratio of red beet level. The addition of red beet appears to increase visceral fat mass and decrease growth, as observed with respect to growth performance parameters (Pinedo‐Gil et al., 2017).

Pinedo‐Gil et al. (2017) reported the inclusion of red beet in the fish diet increased betaine concentration in fish meat compared to the control diet. It is important for the bioactivity of the product that it is interesting to investigate the antioxidant properties that betaine can provide to the final product.

Moreover, the results displayed that the fat content in the fish fillet for 2% treatment was around highest, while the moisture content decreased for 2% treatment to the lowest level (Figure 2). There was no significant difference in fat performance ratio between the 0.5% and 2% treatments on the 42nd day (*p* < .05), while on the 56th day, a significant difference was found between the control and 1% treatments (Figure 5). Figure 7 shows that the condition index for 2% treatment was higher than for 1% treatment on the 56th day. Moreover, the feed conversion ratio for 2% treatment was high, but the highest ratio was found for 1% treatment on the 56th day (Figure 8).

*Beta vulgaris* leaves are widely used in animal nutrition due to their high nutritional value and low price. It seems that different compounds in this plant have positive effects on fish growth. Our findings showed that the condition index in the fish diet was decreased based on the different ratios of *B. vulgaris* leaf powder compared to the control treatment. This result was in agreement with those of Rahimian et al. (2006). It seems that alteration in the taste and abundance of oxalate in *B. vulgaris* leaves is effective in this reduction.

Adding *B. vulgaris* leaf powder to the diet of carp had a positive effect on the nutrition indices, which was in agreement with the results of Reissan‐zadeh et al. (2002). *Beta vulgaris* contains large amounts of carotenoids and vitamin E, which act as preservatives and antioxidants. Apparently, the presence of numerous antioxidants in *B. vulgaris* leaves affects the health of fish. Our findings revealed that the feed conversion ratio increased with adding *B. vulgaris* leaf powder to the fish diet, which was consistent with the results of Reissan‐zadeh et al. (1994). This can be due to a change in the taste of *B. vulgaris* leaf powder. It can be because of the relatively high concentration of oxalate due to inadequate drying of *Beta vulgaris* leaves, which results in poisoning and weight loss (Reissan‐Zadeh et al., 1992).

*Beta vulgaris* is popular for its abundance of a nitrogenous pigment which is water soluble called betalains. Betalains are used in the processing of food products as an additive due to its low toxicity, high water solubility, and natural colorant characteristics. Two major categories of betalains are found to exist in the plant. The first is betacyanin, which is a red pigment. The pigment's major effects are the antioxidant property where it enriches the low‐density lipoprotein which increase the body resistance to oxidation, stimulates the immune system, gives kidney and liver protection, increases cognitive functioning, and guards one's blood vessels against damage and inflammation. *Beta vulgaris* is undoubtedly a very important and underutilized plant worldwide. Its approximate properties have shown that its leaves are a good source of food and animal feed. Most of its medicinal value is due to the phytochemical properties of the plant (Iwuozor & Afiomah, 2020).

Asadi Sharif et al. (2014) reported that the red beet juice powder up to 6% represented good effect on growth performances of Oscar fish. It was found that the most appropriate dose of red beet in the diet for best growth performances of rainbow trout was 4%. These results were agreeing with the results of our study that showing the increase in the percentage of the fish fillet protein had directly proportional to the increase in the concentration of the *B. vulgaris* additive.

### 3.1 Influence of *Beta vulgaris* leaf powder on fillet's nutrient composition

The obtained results for nutrient composition of carp at the end of the experiment period showed that adding 1% of *B. vulgaris* leaf powder to the fish diet decreased the fat content that was significantly different from the other treatments, and was in agreement with the results of Kpogue et al. (2013). The presence of *B. vulgaris* leaf powder in the fish diet increased the fillet protein content, which was in agreement with the results of Reissan‐zadeh et al. (2002). This can be due to the presence of betalains in *B. vulgaris* leaves which was used in lipid metabolism in phosphatidylcholine synthesis and in fatty acid oxidation, reducing the fillet fat. The increase in the fillet ash content can also be for the presence of this plant in the fish diet. This finding was in agreement with the results of Reissan‐zadeh et al. (1994).

More studies have been carried out about the effects of these diets on the fish fillet quality after acute stress (Pinedo et al., 2016). It seems that these results were in agreement with the present study results. Amiri et al. (2019) reported the effects of using *B. vulgaris* pulp in the diet of carp improved the fillet composition. The results showed that adding *B. vulgaris* leaf powder to the fish diet had positive effects on their feed conversion index. Because of the high nutritional value and low price of *B. vulgaris* leaves, they are used to feed animals. The different compounds present in *B. vulgaris* had positive effects on fish growth (Goyal et al., 2015).

The presence of *B. vulgaris* root (10%) in the fish diet not only improved their growth but also enhanced the total carotenoid content and body composition significantly. Therefore, cheap and easily available natural carotenoid sources like beetroot can be incorporated into *B. bendelisis* diet at 10% level to obtain better pigmentation and market value (Nath Jha et al., 2013). The condition factor as growth factor for *Cyprinus carpio* is shown in Figure 7. The condition factor measures for the fish were 0.46, 0.44, 0.47, and 0.62, respectively, on different *B. vulgaris* leaf powder (0.0%, 0.5%, 1%, and 2%) for 28th day. The mean condition coefficients for the *Cyprinus carpio* were 0.46, 0.44, 0.47, and 0.62 and this shows that the fish is in higher average condition than one during the experiment (Ighwela et al., 2011), which indicated good health condition during the experiment and it is indicating an isometric growth, which is the desirable for the fish feed.

## 4 CONCLUSIONS

The present study provided a new perspective on the use of *B. vulgaris* leaf powder in the diet of common carp that improved the fillet composition. The results showed that the addition of *B. vulgaris* leaf powder to the fish diet had a positive effect on their feed conversion ratio. In addition, their protein and fat efficiency ratio were significantly higher than the control treatment. The use of *B. vulgaris* leaf powder (50 mg) in the diet of common carp requires further research. Moreover, determining the effect of *B. vulgaris* leaf powder on fillet compounds, digestive enzymes, and blood components in other important commercial species also needs further studies. In addition, studying the effect of *B. vulgaris* leaf powder on the immune factors of common carp as well as the effect of other doses of powdered *B. vulgaris* leaves on common carp requires further.

## AUTHOR CONTRIBUTIONS

**Ali Aberoumand:** Conceptualization (equal); data curation (equal); formal analysis (equal); funding acquisition (equal); investigation (equal); methodology (equal); project administration (equal); resources (equal); software (equal); supervision (equal); validation (equal); visualization (equal); writing – original draft (equal); writing – review and editing (equal). **Saeed Ziaei‐nejad:** Supervision (equal). **Nahid Amiri:** Formal analysis (equal); investigation (equal); methodology (equal).

## FUNDING INFORMATION

This work was supported by Behbahan Khatam Alanbia University of Technology, Behbahan, Iran.

## CONFLICT OF INTEREST STATEMENT

The authors do not have any conflict of interest to declare.

## ETHICS STATEMENT

This study did not involve human or animal testing.

## INFORMED CONSENT

Written informed consent was obtained from all participants.

## Data Availability

Research data are available after request.
